# Behavioral DiverCity: individual differences in behavior change along an urbanization gradient

**DOI:** 10.1093/beheco/araf035

**Published:** 2025-05-04

**Authors:** Laura Gervais, Megan J Thompson, Pierre de Villemereuil, Tracy Burkhard, Céline Teplitsky, Barbara Class, Denis Réale, Anne Charmantier

**Affiliations:** CEFE, Univ Montpellier, CNRS, EPHE, IRD, 1919 route de Mende, 34293 Montpellier, France; Station d’Ecologie Théorique et Expérimentale du CNRS, UAR 2029, 2 route du CNRS, 09200 Moulis, France; CEFE, Univ Montpellier, CNRS, EPHE, IRD, 1919 route de Mende, 34293 Montpellier, France; Département des sciences biologiques, Université du Québec à Montréal, 141 Avenue du Président-Kennedy, Montréal, Quebec H2X 1Y4, Canada; Institut de Systématique, Évolution, Biodiversité (ISYEB), École Pratique des Hautes Études | PSL, MNHN, CNRS, SU, UA, Les Patios Saint-Jacques, 4-14 Rue Ferrus, 75014 Paris, France; Institut Universitaire de France (IUF), France; CEFE, Univ Montpellier, CNRS, EPHE, IRD, 1919 route de Mende, 34293 Montpellier, France; ISEM: Institut des Sciences de l’Evolution de Montpellier ISEM, Univ Montpellier, CNRS, IRD, Pl. Eugène Bataillon, 34090 Montpellier, France; Lewis & Clark College 615 S Palatine Hill Rd, Portland, OR 97219, États-Unis; CEFE, Univ Montpellier, CNRS, EPHE, IRD, 1919 route de Mende, 34293 Montpellier, France; CEFE, Univ Montpellier, CNRS, EPHE, IRD, 1919 route de Mende, 34293 Montpellier, France; Department of Biology, Ludwig-Maximilians-Universität München, Großhaderner Straße 2, 82152 Planegg-Martinsried, Germany; Département des sciences biologiques, Université du Québec à Montréal, 141 Avenue du Président-Kennedy, Montréal, Quebec H2X 1Y4, Canada; CEFE, Univ Montpellier, CNRS, EPHE, IRD, 1919 route de Mende, 34293 Montpellier, France

**Keywords:** among-individual variance, city, coefficient of variation, multiple-spatial scale, repeatability, trait variation

## Abstract

Urbanization is occurring globally at an unprecedented rate and, despite the eco-evolutionary importance of individual variation, we still have limited insight on how phenotypic variation is modified by anthropogenic environmental change. Urbanization can increase individual differences in some contexts, but whether this is generalizable to behavioral traits, which directly affect how organisms interact with, and respond to, environmental variation, is not well known. Here we examined variation across three behavioral traits linked to stress reactivity, anti-predator response, and novelty-coping (breath rate, handling aggression, and exploration behavior) in great tits *Parus major* along an urbanization gradient. We phenotyped > 1000 individuals across 9 yr, to test whether individual differences in behavior increased with urbanization and spatial environmental heterogeneity. We used two different approaches: a city vs. forest comparison (ie a binary descriptor) and an urbanization gradient approach (ie a continuous quantitative score from 0 to 1) to explore the influence of built-up areas at different spatial scales. Our results reveal that urban individuals display more diverse stress-related and anti-predator behaviors (breath rate and handling aggression), yet show more similarity in their exploratory behavior than forest counterparts. However, there was no evidence that individual variation changes along the percentage of built-up areas for any traits. This study suggest that generalizations about how behavioral traits respond to urbanization will differ across behavioral dimensions. In particular, we may expect decreased individual diversity in urban birds for traits related to behavioral response to novelty.

## Teaser

Urbanization has been shown to alter trait means and more recently to affect the variance of life history and morphological traits, for example by increasing diversity in laying dates. However, the influence of urbanization on behavioral variation is not well understood. Using a long-term study of great tits, we found that birds living in the city may behaviorally react to stress in more varied ways, but they tend to show more similar behaviors when faced with new situations.

## Introduction

Environmental change is a widespread process that occurs naturally across space and time, but human-induced environmental change is occurring at an unprecedented scale and speed, posing new challenges to organisms (Vitousek et al. 1997; Merilä 2012; Pelletier and Coltman 2018). One of the main challenges is urbanization, ie the ultimate replacement of natural landscapes by man-made infrastructures (Dansereau 1957), resulting in a variety of artificial environmental alterations, such as increased noise pollution, impervious surfaces or disturbance by human presence (Niemelä et al. 2011). While some organisms struggle in the face of new selective pressures induced by these changes, others survive or even thrive in urban environments by adjusting their phenotype via individual plasticity or genetic evolution (Hendry et al. 2008; Merilä and Hendry 2014).

An increasing number of studies has documented urban-associated phenotypic changes in a variety of taxa and traits (eg pigmentation in Lepidoptera moths, Kettlewell, 1956; beak morphology and vocal performance in house finches *Haemorhous mexicanus*, Giraudeau et al. 2014; toxin tolerance in killifish, Reid et al. 2016). To date, studies of urban-associated phenotypic shifts have mostly reported changes in mean phenotypes. Phenotypic change can occur not only through a shift in mean, but also through shifts in variation, with important implications for eco-evolutionary processes (Sanderson et al. 2023). Indeed, phenotypic variation could drive evolutionary responses to environmental change as it determines the upper limit of genetic variance and is therefore a prerequisite for selection to act and elicit a response to selection. Cities can act as agents of selection (Charmantier et al. 2024), and thus phenotypic variance can itself be shaped by urban environments in addition to other eco-evolutionary processes (eg plasticity, dispersal, (epi)genetic variation, Reed et al. 2011; Des Roches et al. 2018; Draghi 2019). Hence urbanization can alter the mean and variance of phenotypes, and these phenotypic changes may in turn have multiple consequences for population demography or community dynamics. For instance, within a given population of predator, individuals may vary in which species they prey on most heavily and they can exhibit different levels of variation in prey choice behaviors, even if their average prey choice remains the same (Bolnick et al. 2011). As a consequence, changes in phenotypic variances can have cascading impacts on population composition, dynamics, resilience, and ecosystem services and sustainability (Sanderson et al. 2023). However, surprisingly little is known about the relationship between urbanization and phenotypic variation. Recent reviews hypothesized that phenotypic variation could increase in urban environments due to multiple non-exclusive mechanisms such as limited dispersal, relaxed or heterogeneous selection, increased exposure to mutagens, or developmental plasticity (Capilla-Lasheras et al. 2022; Thompson et al. 2022). To date, however, fewer than ten studies have investigated this hypothesis, with only two providing conclusive support (eg meta-analysis on variance in morphology in great tits *Parus major* and blue tits *Cyanistes caeruleus*; Thompson et al. 2022; and life-history traits in bird species globally, n = 35 species; Capilla-Lasheras et al. 2022).

Urbanization imposes new challenges requiring behavioral changes, such as collecting environmental information in artificial or fragmented habitats, avoiding human disturbances and new predators, or adopting novel foods (Sol et al. 2013). Consequently, certain behavioral traits are particularly well-suited to urban life (Møller 2008; Lowry and Wong 2013; Sol et al. 2013) and show marked divergences between urban and non-urban environments. In particular, urban organisms tend to be bolder, more aggressive, more exploratory, and to tolerate higher levels of disturbance than their non-urban counterparts, which may provide advantages for successful colonization and establishment in new environments (Candolin and Wong 2012). Despite the abundant studies exploring behavioral shifts in response to urbanization, few have examined how urbanization affects behavioral variation (n = 24, published between 2010 and 2022, see Burkhard, Dochtermann and Charmantier (2024) metanalysis for more detail). Recent attempts to tackle this question have compared repeatability, ie the proportion of total phenotypic variation due to among-individual variance (Bell et al. 2009), between urban and non-urban populations. For example, urban-derived speckled wood butterflies (*Pararge aegeria*) raised in a common garden were found to have increased repeatability in boldness (ie latency to approach feeder) compared to rural-derived butterflies (0.50 [0.39 to 0.56] *vs.* 0.15 [0.09 to 0.22]; Kaiser et al. 2019), a result partly explained by both higher among-individual variance and lower within-individual variance in urban-derived butterflies. In contrast, repeatability of boldness in song sparrows (*Melospiza melodia*) did not differ between urban and rural habitats (repeatability of 0.24; Fossett and Hyman 2021); here, however, among- and within-individual variances were not reported, rendering comparison of phenotypic variation between the two habitats difficult. Decomposing repeatability into its components—and reporting these components—is crucial to understanding how phenotypic variation is affected by eco-evolutionary processes: when reported alone, repeatability can be misleading as similar repeatability ratios does not equate to similar among- and within-individual variances (Dochtermann and Royauté 2019). Hence, as repeatability is often reported without the underlying variance components, we still know little about the effects of urbanization on behavioral variance, despite some studies comparing repeatability between urban and non-urban populations.

First, difference in repeatability can result from difference in among-individual variance. In the literature, urban dwellers have been shown to have higher among-individual variance in several ecologically relevant behaviors, including vigilance, aggression, and boldness (eg in woodchucks (*Marmota monax*); Lehrer et al. 2012 or shrews (*Crocidura russula* & *Sorex araneus*); von Merten et al. 2022). Higher among-individual variance in urban populations can reflect underlying differences in genetic variances, eg due to different heterogeneous selection across urban and non-urban habitats (  Hedrick 1986; Barrett and Schluter 2008). Alternatively it can result from lower canalization during development in urban environments in response to the environments experienced during in early life leading to permanent differences across individuals (Kristensen et al. 2018; Lazić et al. 2015; Lindström 1999; see Thompson et al. 2022 for an exhaustive review of putative mechanisms). Higher urban among-individual variance can buffer urban populations from new or fluctuating selective pressures by increasing the likelihood that certain behaviors are well-suited to novel challenges (ie the ‘skill pool effect,’ Giraldeau 1984). Second, though not mutually exclusive, difference in repeatability can also result from differences in within-individual variation, partly as a result of individual behavioral plasticity. Urban dwellers can have greater behavioral plasticity (Sol and Lefebvre 2000; Hendry et al. 2008; Dammhahn et al. 2020), which should help them adjust quickly to novel challenges in the city and, in some cases influence adaptive evolution (Caspi et al. 2022). In short, both among and within-individual components are likely to play an important role in responses to urban environments (Lowry et al. 2013; Sol et al. 2013), hence, examining how urbanization impacts variation in behaviors known to influence fitness would allow a more comprehensive view on the processes that impact urban populations.

Here, we explore how among- and within-individual variance in behaviors change along an urbanization gradient. To do so, we use a long-term study of great tits living along an environmental gradient from natural oak forest to highly urbanized areas. We investigate phenotypic variation in three behavioral traits hypothesized to be involved in how organisms cope with urban environments (Burkhard et al. 2024, , Møller 2010; Atwell et al. 2012): aggression reflecting anti-predator responses (using handling aggression as a proxy), response to acute stress (using breath rate during handling as a proxy), and novel- or challenging-situation coping (using exploration behavior in a novel environment as a proxy, Dingemanse et al 2002; Stubber et al 2013). Previous research on the same study system has shown that urban great tits are more aggressive, faster explorers in a novel environment, and have higher breath rates than those from forest habitats. Interestingly, although these urban phenotypes could help exploiting novel resources, recent selection analyses revealed that they were in fact selected against, associated with decreased survival in both urban and forest environments (Caizergues et al. 2022). It remains unclear whether these documented shifts in the mean value of these behaviors might be coupled with greater behavioral diversity in urban settings.

In this study system, urban great tits show slightly reduced gene flow compared to forest areas, with some genomic evidence of local adaptation which could promote differences in phenotypic variation between habitats (Perrier et al. 2018). We test the recently proposed hypothesis (Thompson et al. 2022) that phenotypic variance should be higher in the most urbanized (prediction 1) and spatially heterogeneous (prediction 2) environments. We aim to determine whether differences in phenotypic variance are due to among-individual variance, within-individual variance, or both. We use two different approaches: a city vs. forest comparison to allow comparison with recent literature, and an urbanization gradient approach to explore different spatial scales at which urbanization could influence behavioral diversity. In some species with large home ranges, cities could impose high environmental heterogeneity comprising a patchwork of natural and anthropogenic features (eg buildings, green spaces), thus contributing to more spatially heterogeneous habitats compared to natural environments (Cadenasso et al. 2007; Corsini et al. 2019; Alberti et al. 2020). Due to increased environmental complexity, resource variability, and anthropogenic stressors, there should be greater diversity in the composition of great tit individual territories in urban environments. Consequently we expect that due to increased environmental complexity, we will find greater among-individual variance in stress-response, aggressiveness and exploration within sampling locations that are the most urbanized (prediction 3) or have the highest spatial heterogeneity in urbanization (prediction 4). Finally, Caspi et al. (2022) predict that behavioral plasticity (ie within-individual behavioral variance) should be enhanced in urban environments. However, the empirical literature shows mixed results (eg higher within-individual variation in cities, Dammhahn et al. 2020; or in forests, Prange et al. 2004; or no difference between cities and forests, Sprau and Dingemanse 2017). Therefore, we do not make directional predictions regarding differences in within-individual variation across the urban landscape.

## Materials & methods

### Study system

**Figure 1.**
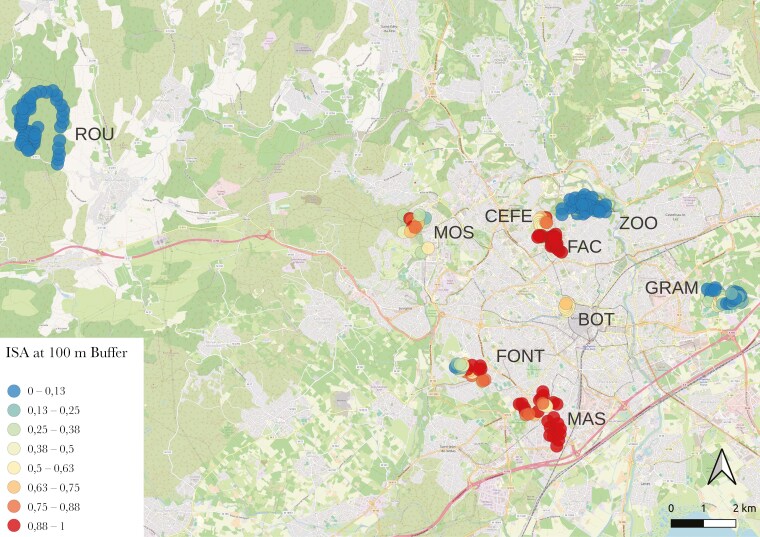
Spatial map of the eight urban locations, the unique forest location and their associated proportion of urbanization at 100 m around each nest-box in the Montpellier area, France. Each point represents a nest-box and is characterized by the average proportion of ISA (ie impervious surface area) illustrated by different colors.

Great tits (*Parus major*) were studied in southern France in La Rouvière (ROU), an oak forest 20 km northwest of Montpellier that has been monitored since 1992 with 230 nest boxes for blue tits (*Cyanistes caeruleus*) and great tits (Blondel et al. 2006). We also monitored tits at eight locations across an urbanization gradient in the city of Montpellier, with around 247 nest boxes monitored since 2011 and hosting mostly great tits (Demeyrier et al. 2016; Charmantier et al. 2017) (Fig. 1 for a spatial overview of the forest location and the eight urban locations).

During the breeding season, nest boxes were visited at least weekly to follow reproduction. Adults were captured in nest boxes when feeding their 10 to 15 d old nestlings. All nestlings and adults were individually ringed with a unique metal ring provided by the French CRBPO (Centre de Recherche par le Baguage des Populations d’Oiseaux) and parents underwent behavioral assays (see below for more details). Behavioral assays were performed on both forest and urban parents captured between 2014 and 2022 (one assay per season for breath rate index and exploration; up to twice per season for handling aggression in case of multiple brooding). The sample sizes vary for breath rate index, handling aggression and exploration score respectively: 760,855, 579 city birds and 299, 411, 233 forest birds. Birds bred 1 to 11 times across the monitoring years: 26% of urban birds and 22% of forest birds had repeated measurements for breath rate index, 23% of urban birds and 18% of forest had repeated measurements for exploration score, and 35% of urban birds and 46% of forest birds had repeated measurements for handling aggression (see Table S1 and S2 for more details on sample sizes).

All protocols were approved by the local ethics committee for animal experimentation of Languedoc Roussillon (CEEA-LR. 05/06/2018) and regional institutions (Prefecture decree no. 2012167-003). The captures were carried out under personal ringing permits issued by the CRBPO for the research ringing program number 369.

### Behavioral assays and description

Once a bird was captured in its breeding nest box, we assessed two reactions to the stress of being handled. First, we immediately recorded its handling aggression (HA) score as soon as we removed the bird from the nest box. Handling aggression reflects aggressive behavior in response to manipulation by potential predators (ie humans) and could serve as a proxy for anti-predator behavior. The bird was held with its face away from the observer and provoked with a finger of the free hand following a standard procedure where the finger approached the bird’s beak three times without touching it. The observer assigned a score ranging from 0 (unresponsive bird) to 3 (aggressive bird spreading wings and tail) in increments of 0.5 following a standardized protocol (see Figure S2A in Caizergues et al. 2022 and Table S1 in; Dubuc-Messier et al. 2017). Immediately after the handling aggression test, the bird was isolated in a cloth bag for 5 min for a standardized period of rest. Following these 5 min, the bird was removed from the bag and held on its back by the handler, who measured its breath rate index (BRI). From 2013 to 2016, breath rate index was estimated as the number of chest movements during 30 s, whereas since 2017, the protocol was updated to measure the time to complete 30 chest movements (Caizergues et al. 2022, Figure S2B). Measurements from 2013 to 2016 were therefore converted to approximate the time required to complete 30 chest movements to obtain the measurements on the same scale. Breath rate index was taken consecutively twice in a row and averaged across the two measures. We consider breath rate index a behavioral trait because breath rate reflects both the physiological function of respiration (ie O2 and CO2 exchange) and respiratory behavior (ie breath rate can be altered by classical and operant conditioning, Ley 1994). Breath rate index correlates with heart rate under restraint (Dubuc Messier et al. 2017) and is often used as a proxy for acute stress response (Carere and van Oers 2004; Krams et al. 2013), with a lower breath rate index (and therefore faster breath rate) reflecting a greater stress response. Finally, the bird underwent an open field test using an open field cage with similar dimensions as in Stuber et al. (2013), to evaluate its exploration behavior in a novel environment (Stuber et al. 2013; Caizergues et al. 2022) which is closely linked to novelty-coping and resource acquisition (Toscano et al. 2016). The bird was placed in an acclimation compartment adjacent to the main open-field cage for 2 min before being released into the exploration room. The videos were analyzed using the BORIS software (Friard and Gamba 2016) to generate an exploration score (ES) by counting the number of flights and hops during the 4 min exploration trial. For a detailed protocol see Charmantier et al. (2017) and Caizergues et al. (2022). Note that these three behaviors have previously been shown to be uncorrelated among and within individuals in the same great tit populations, although with 6 yr instead of 9 yr sampled (Caizergues et al. 2022).

### Quantification of urbanization at different spatial scales

We quantified the degree of urbanization at each nest box where at least one parent was captured (N = 301) using the proportion of impervious surface area (ISA), defined as sealed non-natural surfaces (eg roads, railways, buildings), using the imperviousness density raster datasets from the Copernicus on-line database (resolution 10m. tiles: E38N22 & E38N23. Projection: European Environment Agency 2020; LAEA EPSG 3035). Impervious surface area has previously been shown to correlate with other urban factors such as high temperature (Diamond and Martin 2020), high noise and light pollution, low tree cover, and short distance from roads (Szulkin et al. 2020). The spatial scale at which environmental urbanization impacts organisms is rarely known and may vary across focal traits (Uchida et al. 2021; Waterschoot et al. 2023) hence we quantified the proportion of ISA around each nest box at three different spatial scales: 100, 250, and 1000 meters (Figure S1 for an example of the different buffers). We chose this range to explore effects of urbanization at small, medium, and large spatial scales on behavior, as great tits can have extensive natal dispersal (around 900m on average in females, Dingemanse et al. 2003; Garant et al. 2005; Szulkin and Sheldon 2008), can cover larger areas outside of the breeding season (eg average 600m, max 1800m; van Overveld et al. 2016), yet tend to have smaller home ranges during breeding (approx. 60 to 160 m, Wilkin et al. 2006; van Overveld et al. 2015). Using circular radius buffers at these spatial scales in QGIS (v3.22.0; QGIS Development Team 2022), we counted the number of pixels associated with impervious surfaces and calculated an ISA proportion index (range = 0 to 1. Where 1 = all ISA) around each nest box by dividing by the total number of pixels within each buffer. When considering all nest boxes together, the amount of urbanization correlated moderately between the three spatial scales (rho > 0.75), with most discrepancy at nest boxes in the middle or at the edges of urban parks (Fig. 1). We classified sampling locations as forest if the mean ISA measurements at 1000m were below 5% (ROU) and urban if they were above 5% (CEF, BOT, MOS, MAS, FONT, GRAM, FAC, ZOO, Fig. 1). The mean proportion of ISA around each forest nest box was zero at 100 and 250 meters and 0.0007 at 1000 meters, while the mean proportion of ISA around each urban nest box was 0.48, 0.51, and 0.53 at 100, 250, and 1000 meters, respectively, and ranged from 0 to 1 (see Table S3 for more details for each sampling location and Fig. 1). To assess spatial heterogeneity within the city, we also calculated the within-site variance of ISA (Table S3).

### Statistical analysis

We investigated differences in phenotypic variances between urban and forest habitats across the three behavioral traits which are known to be repeatable, not correlated, and have habitat-specific means (ie urban *vs.* forest mean, Caizergues et al. 2022). First, we conducted a statistical power analysis to assess the support for both among-individual and within-individual variance, indicating how inconsistent the observed effect size is with a scenario of no variance between or within individual (Pick et al. 2023; see Text S1). Then, for each trait, we used a Bayesian generalized linear mixed effects model (GLMM) that allowed the phenotypic mean, among-, and within-individual variances to differ between habitats (also known as heterogeneous variance model, Gianola 1986). We chose the error distribution to fit each trait, ie Gaussian for breath rate index, threshold for handling aggression, and Poisson for exploration score. We ensured that effective sample sizes for each model were higher than 1000. We assessed the convergence of all parameters graphically as well as using the Heidelberger and Walch test of the “coda” package (Plummer et al. 2006). Finally, we graphically controlled the residual assumptions with diagnostic.mcmc from the MCMC.qpcr package (Matz et al. 2013) when residuals were not fixed in the model.

#### Comparing city and forest variance components

To assess whether phenotypic (prediction 1) and among-individual variances (prediction 3) were higher in urban than in forest habitats, we first ran a heterogeneous variance model with two habitat categories (ie two separate random intercepts for urban and forest groups of individuals). We estimated the phenotypic mean, among-individual (Vi), annual (Vy) and within-individual variances (or residual variance, Vr) for each habitat and their corresponding 95% credible intervals (CI). Note that within-individual variance represents the variance among observations of the same individual, and can comprise both plastic responses to unexplained environmental effects and measurement error. We included individual identity and year as random effects with heterogeneous variances across random effects and residual error (model a). For all traits we included an interaction between habitat (urban/forest) and other fixed effects known to influence the focal traits: sex, age (adult *vs.* juvenile) (Charmantier et al. 2017; Caizergues et al. 2021, 2022), date (as the number of day since the 1^st^ January of the year) and the quadratic effect of decimal hour of measure since behavior and metabolism can change throughout the day (Caizergues et al. 2020, 2022). Additionally, to account for possible habituation to multiple captures or tests, we included assay rank (ie number of previous assays, with a value of zero for the first assay) as a continuous fixed effect. As the protocol for breath rate index changed during the study (see Caizergues et al. 2022), we included protocol type as a fixed effect for this trait. Finally, for breath rate index and handling aggression, we accounted for among-observer variance by fitting observer identity as a random effect and included heterogeneous variance for each habitat like the other random effects. As among-observer variance is not a source of biological variance and that we are interested in biological variance we did not include it in the total phenotypic variance estimate for the main analysis reported (but see the legend of table S5). Thus, we estimated the total phenotypic variance for each habitat type as Vp = Vi + Vy + Vf + Vr, where Vf is the variance in biologically relevant fixed effects only (ie sex and decimal hour of the day linked to circadian rhythm, in our specific case, de Villemereuil et al. 2018).

Phenotypic means were highly negatively correlated to trait variances: ρ = −0.72,p-value = 0,06, 0.8, p-value = 0,03, and −0.92, p-value = 0,003 for breath rate index, handling aggression, and exploration score, respectively. Hence we chose to estimate mean-standardized variances (ie coefficient of variation, hereafter CV) to explore patterns in variance independent from the previously described differences in mean (Nakagawa and Schielzeth 2012). Note that CV allows direct comparisons of traits measured on different scales and populations. CV is not generally applied to traits that are not on a ratio or log-interval scale (Pélabon et al. 2020), such as handling aggression. However because the phenotypic mean of handling aggression is different from zero, the CV is interpretable in our specific case, but not comparable to other traits or studies. To estimate the phenotypic mean, marginalized across sex and age we used the posterior distributions of predictions (Table S4). To compare variances between urban and forest birds, we estimated the natural logarithm of the ratio between the coefficients of variations from urban and forest (ie lnCVR,  Nakagawa et al. 2014) and its 95% credible interval, such that lnCVR = log(CV_urb/CV_rur) for each variance component (lnCVR_P, lnCVR_I, lnCVR_R, lnCVR_Y for total phenotypic, among-individual, within-individual or residual, year components respectively). Traits with higher variation in urban habitats will have a positive lnCVR, traits with lower variation in urban habitats will have a negative lnCVR, and the lnCVR will be zero when the variation is similar in both habitats, We also estimated adjusted repeatability rpt = Vi/Vp and tested differences in repeatability by calculating the log repeatability ratio lnRPT = rpt_urb/rpt_rur to allow comparisons to similar estimates in the literature. We interpret lnCVR and lnRPT (ie effect sizes) as evidence for a difference between urban and forest when the 95% CI does not cross zero. To compare mean behaviors between urban and forest habitats, we computed log response ratios (lnRR = log(mean_urb/mean_rur); Nakagawa et al. 2014).

#### Phenotypic variance across the urban gradient

*T*o investigate whether total phenotypic variance and among-individual variance within the city increased with urbanization (prediction 1 and 3) and spatial heterogeneity (prediction 2 and 4), we ran two-step models. First, we estimated mean-standardized among-individual variance for each location by running a heterogeneous variance model (model b). For each trait in this model, we estimated variance components separately for each of the 9 locations (ie nine separate random intercepts grouping individuals by sampling location). This model had the same random and fixed effects as described for model a, but we removed the interaction term between habitat and sex, age, and the quadratic effect of decimal hour, to avoid over-fitting the model. We also fitted homogeneous instead of heterogeneous variance structures for the year and observer random effects as there was no evidence that these variance components differed between urban and forest habitats (Breath rate index: LNCVR_Y = −1.13[−2.4;0.26]; handling aggression: −0.04[−0.83;0.78]; exploration score: 0.94[−0.84;3.74]) or observer (breath rate index: LNCVR_O = −0.50[−1.53; 0.42]; handling aggression: −0.22[−0.91; 0.50]).

Second, we estimated the strength and direction of the association between the mean-standardized phenotypic or among-individual coefficients of variation (CVP and CVI respectively, from model b mentioned above) with mean ISA and variance ISA (ie spatial heterogeneity) at each sampling location. As the means and variances of ISA were on different scales, we centered and scaled them: (x—mean(x))/sd(x), where × is mean or variance ISA. Two locations within the city had less than 30 observations and high uncertainty around the variances of model b mentioned above, so we decided to exclude these locations (CEF and BOT, Table S2, note that conclusions were not sensitive to their inclusion) and used the remaining seven urban locations for this analysis. Finally, we ran a Bayesian regression model on the mean-standardized posterior variance estimated within each iteration of model b, thus generating the uncertainty around the phenotypic mean and variance components. We included mean ISA and variance in ISA as fixed effects, both measured on the same spatial scale. Mean and variance ISA were not colinear as the absolute values of the correlations between both variables were well below 0.8 (Young 2018) (ρ_spearman_ = −0.12, p-value = 0.793; −0.57, p-value = 0.15; and −0.26, p-value = 0.53 for 100, 250, and 1000m scales, respectively). We used each iteration from model b to run these new models (one model per iteration of model b) with the three different spatial scales of ISA independently. We checked the results with and without the forest locations to ensure that the forest data did not drive the correlation alone.

To determine which spatial scale was the most relevant, we investigated which spatial scale of urbanization explained the most variance in among-individual variation (ie *“*the scale of effect,” Martin and Fahrig 2012) to provide insight into the relevant scale for each trait and variance components. First, we calculated each model’s fit by estimating a Bayesian-R², the variance of the predicted values divided by the variance of the predicted values plus the expected variance of the errors (Gelman et al. 2019). We then averaged the estimates across the different models generated at each spatial scale. We ran the same models as described above for breath rate index and exploration score within-individual variance. Handling aggression had no residual variance; thus, we did not calculate within-individual variation. For breath rate index, the intermediate scale (250m) explained the most variance in phenotypic variation (R-squared = 0.41) and among-individual (250m, R-squared = 0.31), while the 1000m scale explained the most variance in within-individual variation (R-squared = 0.42) (see Figure S2, S3, S4 for the other spatial scales). For handling aggression, the smallest spatial scale (100m) explained the most variance in both phenotypic and among-individual variation (R-squared = 0.52 and 0.48 for phenotypic and among-individual variation respectively). Finally, for exploration score, the 100m scale explained the most variance in phenotypic variation (R-squared = 0.35), while the largest spatial scale (1000 m) explained the most variance in both among-individual and within-individual variation (R-squared = 0.63 and 0.49 respectively). We report model results only for the spatial scales at which urbanization explains the most behavioral variation; results for all other spatial scales can be found in the supplementary materials online (Figure S2, S3, S4).

The analyses for models a and b were conducted using the MCMCglmm package (Hadfield 2010) with default priors. For our last analysis, the model was run on the posterior distributions generated from the MCMCglmm (model b), independently utilizing the rstanarm package (Goodrich et al. 2024), which allows to run linear-regression models. The analyses were performed on R version 4.3.0 (R Core Team, 2021, released on 2023-04-21).

## Results

### Is phenotypic variation higher in more urbanized sites (Prediction 1)*?*

**Figure 2.**
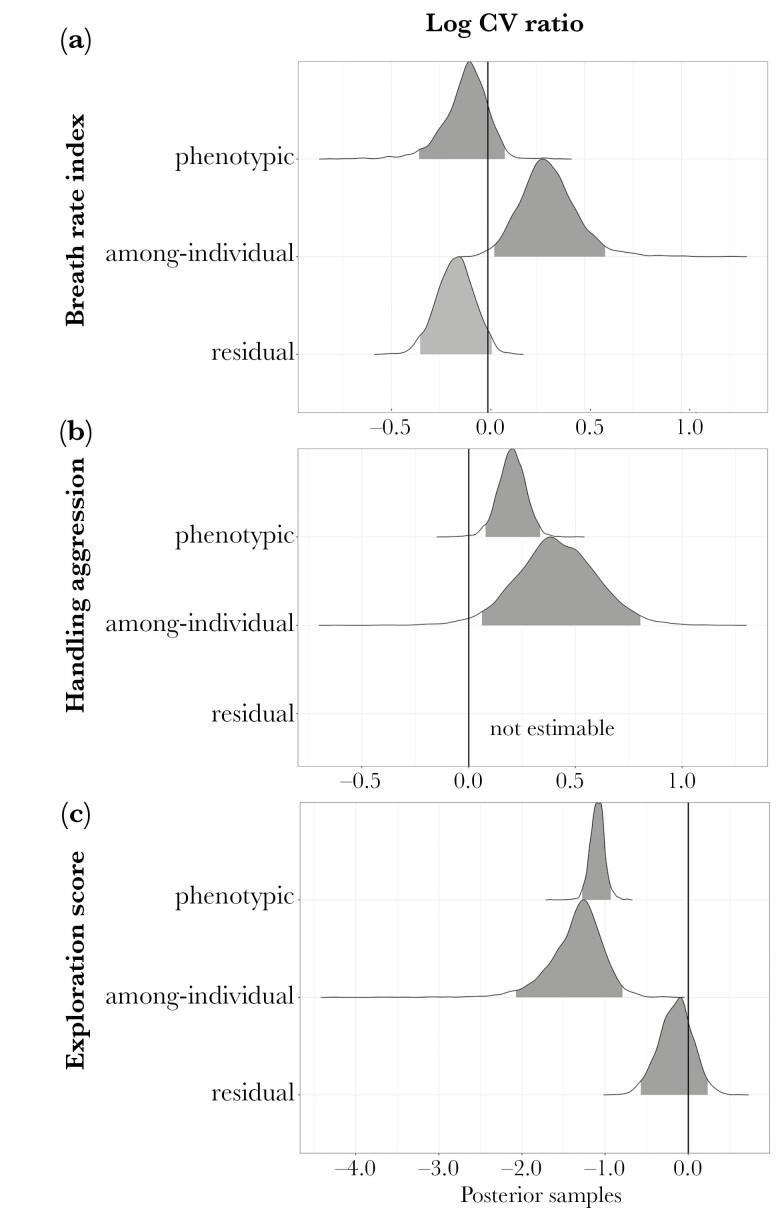
Comparison of phenotypic variation in three behaviors in urban and forest great tits. The comparison of phenotypic variation is represented by the posterior samples of the log-coefficient variance ratio (lnCVR) for behavioral traits (A: Breath rate index, a proxy for stress response; B: Handling aggression, a proxy for anti-predator behavior; and C: Exploration score, a proxy for novel-coping behavior) in great tits captured in forest vs urban environments in and around Montpellier, France. From top to bottom in each panel: lnCVR for total phenotypic, among-individual and within-individual variance (residual). Traits are more variable (higher log CV ratio) in urban habitats when estimates are positive (ie right of the solid black line). In gray is the 95% credible interval of the posterior distributions.

**Figure 3.**
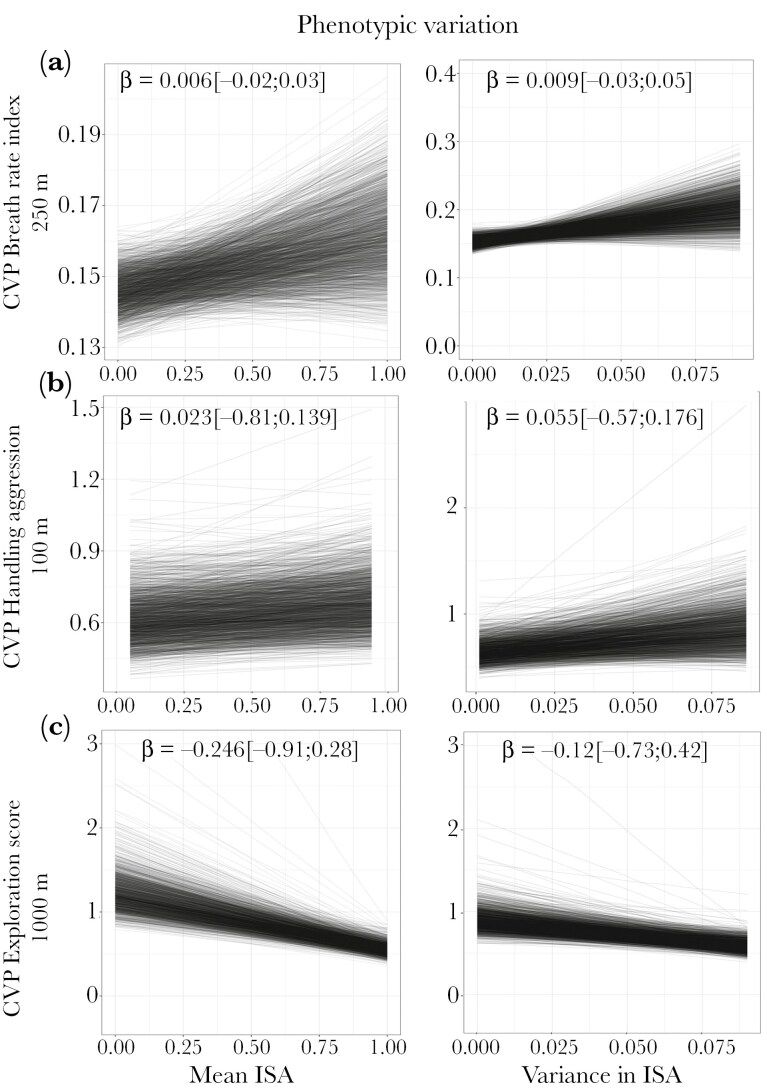
Relationship between measures of urbanization and phenotypic variation in A) breath rate index, B) handling aggression, and C) exploration score. Left, predicted mean-standardized phenotypic variance (CVP) and mean impervious surface area (ISA); right, CVP and variance in ISA. Each behavior’s relevant “scale of effect” (spatial scale that explained the most variance in phenotypic variation) is indicated on the y axis. CVP depicted here were estimated from the posteriors distributions of model b. Each line represents the predictions of one Bayesian model (one model per iteration of model b). Note that CVP are expressed on the latent-scale for handling aggression and exploration score. β is the coefficient of relation between CVP and mean/variance ISA when combining the 1000 posterior distributions into one and is highlighted in bold when the 95% credible interval does not overlap zero. Please see Figure S3 for the full distributions of the relationship coefficients across the 1,000 posterior distributions.

Urbanization was associated with phenotypic variation in some, but not all, of the behavioral traits (Fig. 2). These relationships varied both in magnitude and direction and were affected by urbanization metric (categorical vs continuous; see summary Table 1; raw variance can be found in table S5 and S6). We found no difference in phenotypic variation for breath rate index between categorical metrics of urbanization (urban vs forest, Log ratio between coefficients of total phenotypic variation: lnCVR_P = 0.02 [−0.11; 0.16]), a result that was corroborated by our model considering mean impervious surface area (ISA), a continuous metric for urbanization (β_meanISA = 0.006 [−0.003; 0.015]). Conversely, urban birds were 1.27 times more phenotypically variable in their handling aggression than forest birds (lnCVR_P = 0.24 [0.12; 0.35]), though when we considered continuous urbanization, the relationship between urbanization and phenotypic variation disappeared (β_meanISA = 0.023 [−0,081;0,139]. The relationship between urbanization and phenotypic variation was strongest for exploration, though it was counter to our prediction 1: urban birds were 2.97 times less phenotypically variable in exploration (lnCVR_P = −1.09 [−1.26; −0.91]) than forest birds, a finding that was not supported by our continuous urbanization model (β_meanISA = −0.246 [−0,91;0,28], Fig. 3C).

**Table 1.**
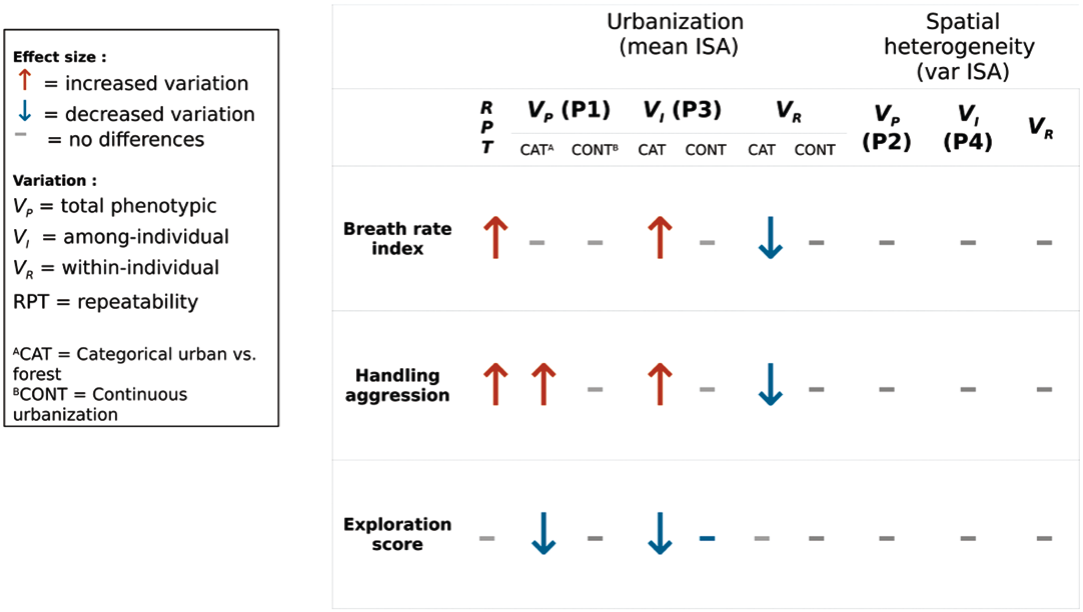
Summary of the results when comparing variance components across city and forest (CAT) or along an urbanization gradient (CONT), according to predictions 1 (P1), 2 (P2), 3 (P3), and 4 (P4).

### Does among-individual variation increase with urbanization? (Prediction 3)

Among-individual variation systematically differed between urban and forest birds for the three behavioral traits though not always in the predicted direction of higher variation in more urbanized sites (Table 1, Fig. 2). Urban birds had 1.35 times and 1.5 times more among-individual variation for breath rate index and handling aggression, respectively (lnCVR_I = 0.3 [0.03; 0.6] and 0.41 [0.061; 0.8], Fig. 2A,B, respectively) but 3.67 times less among-individual variation for exploration (Fig. 2C, lnCVR_I = −1.3 [−2.08; −0.8]) than forest birds. When we considered continuous urbanization, however, the relationship between among-individual variation and urbanization disappeared for breath rate index, handling aggression (β_meanISA = 0.009 [−0.03; 0.05], 0.035 [−0.07; 0.16], respectively) and exploration score (β_meanISA = −0,25 [−0,91;0,28]). Finally, we found evidence that urban birds were more repeatable in both breath rate index (lnRPT = 0.42 [0; 0.95], with 99% of posterior distributions being positive) and handling aggression (lnRPT = 0.45 [0.18; 0.81]), while repeatability of exploration did not differ (lnRPT = −0.12 [−0.41; 0.18]) (Table S5).

### Are phenotypic variation and among-individual variation greater in more heterogeneous sites (Predictions 2 & 4)*?*

**Figure 4.**
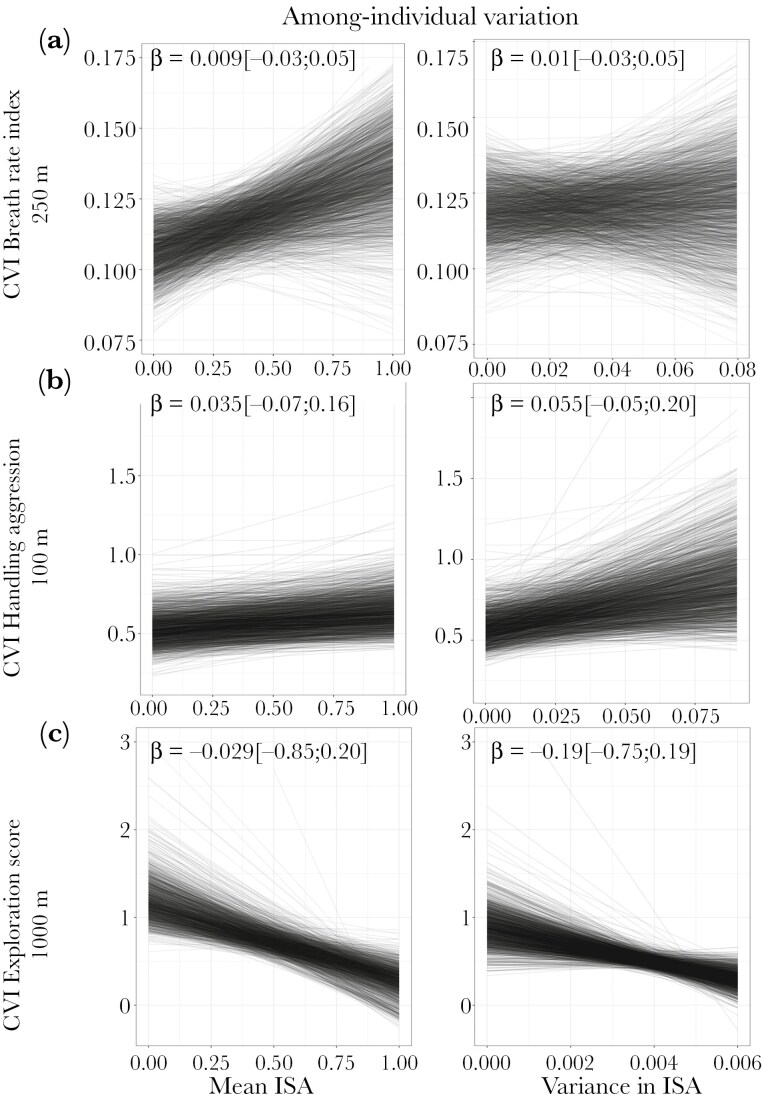
Relationship between measures of urbanization and phenotypic variation in A) breath rate index, B) handling aggression, and C) exploration score. Left, predicted mean-standardized among-individual variance (CVI) and mean impervious surface area (ISA); right, CVI and variance in ISA. Each behavior’s relevant “scale of effect” (spatial scale that explained the most variance in phenotypic variation) is indicated on the y axis. CVI depicted here were estimated from the posteriors distributions of model b. Each line represents the predictions of one Bayesian model (one model per iteration of model b). Note that CVI are expressed on the latent-scale for handling aggression and exploration score. β is the coefficient of relation between CVP and mean/variance ISA when combining the 1000 posterior distributions into one and is highlighted in bold when the 95% credible interval does not overlap zero. Please see Figure S3 for the full distributions of the relationship coefficients across the 1000 posterior distributions.

Variance in impervious surface area, ie *s*patial heterogeneity, was not strongly associated with either greater phenotypic variation (prediction 2) or greater among-individual variation (prediction 4) in breath rate index, handling aggression and exploration score (BRI: β_varianceISA = 0.01 [−0.001; 0.021] and β_varianceISA = 0.009 [−0.03, 0.05]; HA: β_varianceISA = 0.055 [−0,057;0,176] and 0.055 [−0.05; 0.20]; ES: β_varianceISA = −0,12 [−0,73;0,43] and 0,19 [−0,75;0,29] for phenotypic and among-individual variation, respectively; Fig. 3,4 A,B,C).

### Patterns of within-individual variation along the urban gradient

Urban birds exhibited 1.18 times less within-individual variation than forest birds for breath rate index (Fig. 2A), and within-individual variation in this trait linearly. In contrast, we found no difference in within-individual variation in exploration between urban and forest birds (Fig. 2C). There was no relationship between within-individual variation and mean impervious surface or spatial heterogeneity for both traits (BRI: B_meanISA = −0.014 [−0,039;0,012], B_varISA = 0.015 [−0,011;0,042] and ES: B_meanISA = −0,04 [−0,36;0,24], B_varISA = −0,10 [−0,41;0,18], Figure S5A,C).

## Discussion

Recent theoretical and empirical work has hypothesized that phenotypic variation, the raw material for selection, may be higher in urban compared to non-urban populations (Capilla-Lasheras et al. 2022; Thompson et al. 2022), in particular for species with home ranges encompassing large environmental heterogeneity in cities. However, this hypothesis has rarely been investigated in behavioral traits (but see Sanderson et al. 2023; Burkhard et al. 2024). We examined if urban populations of great tits displayed more behavioral variation than forest populations (Prediction 1), and whether this was due to higher among-individual variation (ie differences between individuals, Prediction 3). Our results show that birds in more-urbanized habitats tended to be more variable in their stress response and in anti-predator behavior, yet displayed stronger similarity in exploration (Fig. 2).

### Urbanization reduces behavioral variation in exploration

In contrast with our predictions 1 and 3, we found that total phenotypic and among-individual variation in exploration decreased in more urbanized environments, and there was no relationship between phenotypic variation and variance in impervious surface, ie spatial heterogeneity (Figs 3, 4). One possible explanation for this result is that traits strongly linked to fitness may show decreased phenotypic variance if under strong stabilizing selection (Brommer 2011; Thompson et al. 2022; Sanderson et al. 2023). Different facets of exploratory behavior, such as the affinity for exploration and exploration speed (“fast” vs ‘slow’), are closely linked to resource acquisition, habitat expansion, growth rate, and other fitness-related traits, both in great tits (Nicolaus et al. 2012; Toscano et al. 2016; Mouchet et al. 2021; Caizergues et al. 2022) and in other species (eg red squirrels, Santicchia et al. 2018; cane toads, Gruber et al. 2017; brown trout, Adriaenssens and Johnsson 2011). Furthermore, more extreme expressions of exploration—for example, strong aversion to novelty or strong preference for novelty—can be maladaptive (eg Cavigelli and McClintock 2003; Robertson et al. 2013; Mitchell et al. 2016). Birds in urban environments may display less diverse exploration strategies if extreme exploratory phenotypes are selected against.

While we have some understanding on how selection acts on average exploratory behavior, we still lack knowledge regarding its effects on different components of phenotypic variation, in particular variation within and among individuals which in labile traits can both be shaped by linear and non-linear selection (Araya-Ajoy et al. 2023). More generally, the present literature offers scarce knowledge on whether and how selection might differ between urban and forest environments (Charmantier et al. 2024). Investigating whether the intensity and direction of selection act differently on the various variance components in urban versus forest environments would provide a more comprehensive understanding of the eco-evolutionary processes shaping trait diversity in urban populations. Recent work in our populations suggests that while exploration behavior is under stabilizing selection against extreme exploratory phenotypes in urban tits, these selection patterns are not exclusive to the urban environment; indeed, forest populations are under very similar stabilizing selection patterns (Caizergues et al. 2022). Consequently, we posit that the reduced behavioral diversity in exploration observed in our urban populations might not be due to habitat differences in selection. Instead, the decreased variation in urban populations could result from urban individuals uniformly adjusting their behavior to novel stimuli in the same way (thus reducing variance) through habituation (as shown in blue-tailed skinks, Williams et al. 2019), while forest birds exhibit more diverse responses to novel stimuli.

Alternatively, though not mutually exclusively, increased variance in exploration strategies in forest birds could reflect higher intra- or inter-specific competition, which can each drive phenotypic differences (Swanson et al. 2003; Pfennig and Pfennig 2012; Levis et al. 2020). In our study system, nest box occupancy increased as impervious surface decreased (Figure S6), suggesting that breeding density, and hence competition for resources (ie both intraspecific competition and interspecific competition with blue tits, *Cyanistes caeruleus*), may be higher in forested areas. Further research is needed to help link these patterns of behavioral variation with great tit ecology and demography.

### Impervious surface does not predict habitat differences in behavioral variation for aggression and breath rate

In line with prediction 1, we found that urban birds exhibited greater behavioral diversity in breath rate and handling aggression than forest birds, but contrary to our expectation (predictions 2 and 4) this difference was not related to average impervious surface area, nor with spatial heterogeneity in impervious surface. However we only tested for a linear relationship and urbanization proceeds non-linearly, with each patch having its own history ( Ouyang et al. 2018), possibly explaining the discrepancies between the city versus forest and gradient approach. Our results disagree with findings from previous research demonstrating that higher landscape heterogeneity in urban versus non-urban habitats is associated with more variation in life-history and behavioral traits in urban versus non-urban bird populations (Capilla-Lasheras et al. 2022). Habitat artificiality and heterogeneity might not affect among-individual behavioral variation if individuals reduce the environment heterogeneity encountered by choosing habitats that match their behavior (ie matching habitat choice; Holtmann et al. 2017). For example, Carrete and Tella (2009) hypothesize that the distribution of burrowing owls across habitats with varying levels of human disturbance may be influenced by individual habitat selection decisions, which are driven by each owl’s sensitivity to disturbance. In addition, urban environments are characterized by diverse micro-habitats with varying levels of human disturbance, resource availability, light, sound or air pollution, or predator pressure, which might all impact behaviors but not correlate linearly with mean impervious surface nor spatial heterogeneity in impervious surface. Exploring the mean-level and spatial heterogeneity in these urban stressors individually would help identify environmental features shaping behavioral variation in birds and other taxa (Rivkin et al. 2019; Alberti et al. 2020; Szulkin et al. 2020).

Higher phenotypic variation for aggressiveness in the city contrasts with evidence in the literature suggesting that urban individuals tend to be more homogeneous in predator avoidance behaviors (Geffroy et al. 2020). Higher diversity in aggressiveness and breath rate across individuals (ie among-individual variance) suggests that urban great tits may be better equipped to handle novel challenges, such as predators or sources of stress, due to the skill pool effect (ie diversity increases the likelihood that some behaviors are suited to new challenges; Giraldeau 1984). However, both traits are under stabilizing selection in our populations (Caizergues et al. 2022), so higher urban phenotypic variance implies a higher fitness load in the city compared to the forest (Bolnick et al. 2011), which could contribute to a lower population growth rate in the urban environment. Greater among-individual variation in urban behaviors could result from adaptive or maladaptive developmental plasticity in response to spatial environmental heterogeneity or by larger genetic variance in response to fluctuating or relaxed selection (Wolf and Weissing 2010). For instance, reduced predation pressure in urban environments may lead to relaxed selection and increased phenotypic variation in anti-predator behaviors such as aggressiveness (Fischer et al. 2012; Eötvös, Magura and Lövei 2018; Lokatis et al. 2023). Note that while some studies suggest vertebrate predators are more abundant in cities while predation rates decline (Fischer et al 2012), predation risk for adult passerines in cities has not yet been evaluated. Future studies are needed to uncover if higher among-individual variation for aggression and breath rate in the city are characterized by higher or lower among-individual genetic variation. While they are difficult to implement in vertebrates, common garden and quantitative genetic (genomic) approaches may be the most useful opportunities to deciphering the mechanisms underpinning trait variation and further understand how urbanization impacts the ability of species to persist and evolve (Schell 2018).

### Different spatial scales are relevant to explain among-individual variation in different behavioral traits

While the increasing availability of remote sensing data provides a great opportunity to extract environmental heterogeneity at multiple scales (Kuenzer et al. 2014), the spatial scale at which urbanization affects organisms is an important yet still overlooked issue (Moll et al. 2020). Finding the relevant spatial scale of analysis is crucial for understanding the effects of the urban environment on behavioral diversity as estimations of environmental heterogeneity can vary greatly depending on the spatial scale and the environmental features measured. For example, in our case, the 1000-meter scale likely smooths out important environmental differences (as illustrated in Figure S1), such as localized sources of stress, while it may better capture heterogeneity in resource availability if it is representative of the home range of the focal species. Our results showed that the smallest spatial scales explained the most among-individual variance in aggressiveness, but that the opposite pattern occurred for exploration speed. This is in line with previous studies demonstrating that the scale of effect of urbanization is dependent on the trait studied (Martin 2018; Capilla-Lasheras et al. 2022; Waterschoot et al. 2023). The scale of effect for exploration behavior was a buffer radius of 1000 meters around the breeder’s nest-box, which aligns with great tits exploring and foraging at large spatial scales around their nest (approximately 3,500 to 4,000 m2, 95% KDE density, according to Naef-Daenzer 2000, though note that this radiotracking study was done in an oak forest). In contrast, the scale of effect for stress-related and anti-predator behaviors reflected more local impacts of the environment on breath rate (250 m) and handling aggression (100 m). Similar results have been shown in blue tits, where average exploration and handling aggression were influenced by large- and small-scale ecological conditions, respectively (Dubuc-Messier et al. 2017). The 1000-meter scale may indicate a longer-term response of behavioral traits to urban environments, as dispersal and gene flow occur over such large distances and could have long-term effect on phenotypic variance. In contrast, the 100m scale might reflect an acute and more immediate response to specific stressors. The use of remote sensing data opens an exciting avenue for investigating the different temporal and spatial scale effects of urban-driven evolutionary processes, while providing standardized environmental metrics that will allow comparison of effect sizes across studies (Szulkin et al. 2020).

## Conclusion and perspectives

Our findings reveal a complex scenario wherein urban birds exhibited higher among-individual variance in anti-predator and stress-related behaviors but lower diversity in exploratory behavior, compared to forest birds. These results imply reduced opportunity for selection on novelty-related behaviors in the urban context, but an increased opportunity for selection on predator and stress-related behaviors, providing a foundation to understand the largely overlooked relationship between urbanization and trait variance that might have profound effects on eco-evolutionary dynamics. Note that the historical nest box setup in our study locations based on one relatively homogeneous forest location versus multiple urban locations represented an inherent limitation that calls for further comparisons across multiple forest and city study areas. The three behavioral traits studied here are under stabilizing viability selection, implying that the described differences in variance could have profound consequences for population dynamics. While we lack knowledge on the relative contributions of environmental and genetic factors to the documented variance differences, further studies are needed that combine fitness consequences and the genetic basis of such behaviors along an urban gradient to fully understand the impact of urbanization on ecological and evolutionary predictions.

## Supplementary Material

araf035_suppl_Supplementary_Materials_1

## Data Availability

Analyses reported in this article can be reproduced using the data and R-scripts provided by the authors https://doi.org/10.5061/dryad.c866t1gjp.
