# Supplementary material for: Behavioral DiverCity: individual differences in behavior change along an urbanization gradient
Source: Behav Ecol. 2025 May 4;36(4):araf035. doi: 10.1093/beheco/araf035 (PMC12202043; doi:10.1093/beheco/araf035)
Supplement: araf035_suppl_Supplementary_Materials_1 [file araf035_suppl_supplementary_materials_1.docx]

# Supplemental files


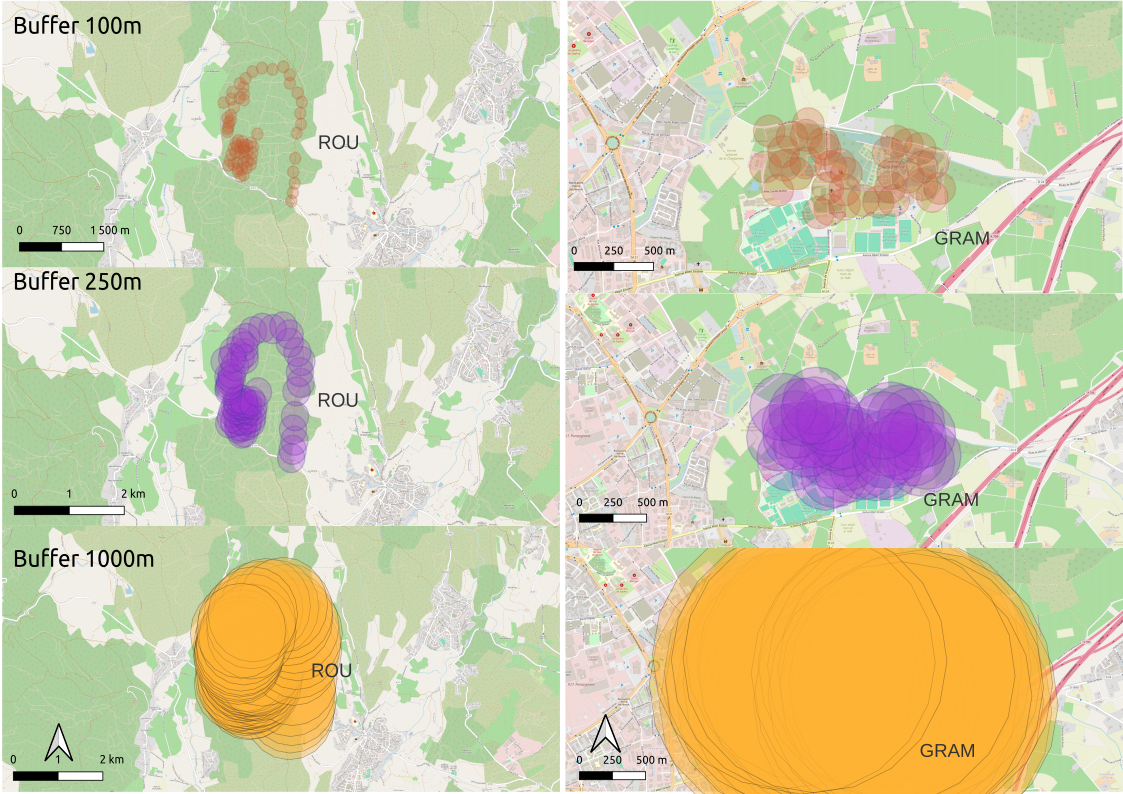


**Figure S1 - Different spatial scales used to assess urbanization around each nest box for the forest site on the left and one urban site on the right.** We used three different buffer radii: 100m (in red), 250m (in purple), and 1000m (in yellow).

**Table S1. Summary statistics on the raw data for three behavioral traits analysed in this study**

| **Traits** | **Metrics** | **Urban** | **Forest** |
| --- | --- | --- | --- |
| **Breath rate index** | Number of individuals | 760 | 299 |
|  | Number of observations | 1041 | 379 |
|  | Mean number of obs/individual | 1.53 | 1.31 |
|  | Sample size for each number of measurements (e.g. 565 individuals measured only once in the city). | 1 :565  2:127  3:42  4:13  5:9  6:2  7:1  8:0  9:0  10:0  11:1 | 1:233  2: 54  3: 8  4: 3  5: 1  6: 0  7: 0  8: 0  9: 0  10: 0  11: 0 |
|  | Raw Phenotypic mean (sd) | 13.44  (2.23) | 14.14  (2.23) |
| **Handling aggression** | Number of individuals | 855 | 411 |
|  | Number of observations | 1327 | 748 |
|  | Mean number of obs/individual | 1.61 | 1.77 |
|  | Sample size for each number of measurements (e.g. 559 individuals measured only once in the city). | 1:559  2:179  3:61  4:39  5:9  6:3  7:4  8:1 | 1:222  2:105  3:44  4:25  5:9  6:4  7:1  8:1 |
|  | Raw phenotypic mean (sd) | 1.75 (1.09) | 1.73(0.97) |
| **Exploration score** | Number of individuals | 579 | 233 |
|  | Number of observations | 751 | 282 |
|  | Mean number of obs/individual | 1.31 | 1.19 |
|  | Sample size for each number of measurements (e.g. 446 individuals measured only once in the city). | 1:446  2:98  3:26  4:8  5:1 | 1:191  2:35  3:7  4:0  5:0 |
|  | Raw Phenotypic mean (sd) | 59.74(62.65) | 29.42(50.78) |

**Table S2. Number of observations per location (9 locations) and sex for each traits BRI (Breath rate index),HA (Handling aggression) and ES (Exploration score).**The 9 locations are ordered by ascending mean ISA (buffer 1000m).

|  | **rou** | **gram** | **zoo** | **mos** | **font** | **cef** | **fac** | **mas** | **bot** |
| --- | --- | --- | --- | --- | --- | --- | --- | --- | --- |
| **BRI female** | 238 | 115 | 178 | 45 | 79 | 14 | 49 | 81 | 11 |
| **BRI male** | 148 | 103 | 163 | 41 | 69 | 11 | 39 | 81 | 10 |
| **HA female** | 409 | 137 | 188 | 69 | 101 | 14 | 60 | 100 | 15 |
| **HA male** | 367 | 132 | 209 | 61 | 95 | 10 | 50 | 101 | 13 |
| **ES female** | 128 | 77 | 95 | 35 | 48 | 6 | 25 | 60 | 7 |
| **ES male** | 99 | 69 | 94 | 31 | 42 | 4 | 20 | 60 | 6 |

**Text S1 : Statistical Power analysis and Relative bias estimates of variance components.**

To ensure our sampling (both in terms of the number of individuals and repeated observations) was sufficient to assess support for both among-individual and within-individual variance, and to accurately estimate the variance components, we conducted simulation analyses following the approach of Pick et al. (2023). We used the squidSim R-package to simulate a population based on our data structure (e.g., number of individuals and repeated measurements, number of years, and observers) and trait distribution (e.g., Gaussian for BRI and Poisson for ES) for urban and forest populations separately, according to the following variance component

VI (among-individual) = 0.3,
Vyear (year) = 0.05,
Vobs (observer) = 0.02,
Vr (within-individual/residual) = 0.63,

so that total phenotypic variance (VP) equals one, and thus, repeatability equals 0.3. We created 1,000 datasets and ran a linear mixed model for each of these datasets, based on the same model structure as our main analysis. Based on these 1,000 models, we estimated the relative bias as :
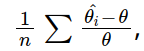
where θ is the true value, θi is the model estimate from ith simulation in a parameter set and n is the number of simulations (Pick et al 2023). In addition, to assess the statistical power to detect variance components different from zero, we simulated 1,000 null distributions (i.e., datasets where the simulated variance of interest is equal to zero) following Pick et al. (2023) and using the squidSim R-package. The variances used to simulate the null distribution are the same as those estimated for our analysis (Table S4). The only exception is that we defined VI=0 and VP=VI_actual_+Vr_actual_+Vf_actual_ (where VI, Vf, and Vr are the among-indivudal, fixed-effect and residual variances estimated and reported in Table S3), to test the statistical power to support VI. Then, we tested the statistical power to support Vr, by defining, VP=VI_actual_+Vr_actual_+Vf_actual_ and Vr=0. Based on these 1,000 null distributions (1000 to test VI and 1000 to test Vr), we estimated statistical power as the percentage of the actual estimates that were greater than median value of the posterior distributions from the null distributions (VI_actuall_>VI_null_ and Vr_actual>_Vr_null_) (Pick et al., 2023).

Note that the squidSim R-package does not allow the simulation of categorical traits (i.e., like our handling aggression traits). However, since we have a similar data structure for handling aggression than for the other traits, we believe we will have similar statistical power and relative bias in the estimation of variance.

Results of the simulations :

We have high statistical power to detect variance components different from zero for exploration score (power = 100% for Vi and Vr) and showed relatively weak bias of -8.98% and 8% for Vi and Vr, respectively, in the urban context. For the forest dataset, the relative bias of exploration variance was -10.62% and 11% for Vi and Vr, respectively (power = 100% for both Vi and Vr). We also have high statistical power to detect variance components different from zero for the breath rate index (power = 100% for Vi and Vr) and showed relatively weak bias of -1.2% and 1.1% for Vi and Vr, respectively, in the urban context, and -8.3% and 3.69% for Vi and Vr, respectively, in the forest context.

**Table S3. Mean and variance of proportion of impervious surface area (ISA) at 100, 250 and 1000 meters around nest-boxes per sampling location (1 rural location ROU and 8 urban locations).** Locations are ordered by ascending proportion of urbanization within a 1000-metre buffer around the nest.

|  | **100 meters** | | **250 meters** | | **1000 meters** | |
| --- | --- | --- | --- | --- | --- | --- |
| **Locations** | **Mean** | **Variance** | **Mean** | **Variance** | **Mean** | **Variance** |
| **ROU** | 0.000 | 0.000 | 0.000 | 0.000 | 0.001 | 0.000 |
| **GRAM** | 0.177 | 0.028 | 0.171 | 0.017 | 0.185 | 0.002 |
| **ZOO** | 0.049 | 0.002 | 0.092 | 0.005 | 0.303 | 0.003 |
| **MOS** | 0.565 | 0.07 | 0.547 | 0.026 | 0.499 | 0.003 |
| **FONT** | 0.61 | 0.09 | 0.682 | 0.05 | 0.633 | 0.005 |
| **CEF** | 0.669 | 0.025 | 0.709 | 0.001 | 0.671 | 0.001 |
| **FAC** | 0.975 | 0.001 | 0.931 | 0.002 | 0.826 | 0.001 |
| **MAS** | 0.903 | 0.022 | 0.9 | 0.005 | 0.83 | 0.005 |
| **BOT** | 0.557 | 0.008 | 0.818 | 0.000 | 0.933 | 0.000 |

**Table S4 - Predicted phenotypic mean per location and their associated 95% credible interval based on a model accounting for sex, age, number of captures, decimal hour of measure, julian date and methodological as fixed-effects.** Locations are ordered by ascending proportion of urbanization within a 1000-metre buffer around the nest.

| **Locations** | **Breath rate index** | **Handling aggression** | **Exploration Score** |
| --- | --- | --- | --- |
| **ROU** | 14.1[13.5-14.6] | 4.00[2.0-6.0] | 1.34[0.824-1.83] |
| **GRAM** | 13.2[12.7-13.8] | 4.6[2.5-7.0] | 2.2[1.7-2.7] |
| **ZOO** | 13.5[12.9-14.0] | 4.0[2.1-6.2] | 3.1[2.7-3.6] |
| **MOS** | 13.8[13.1-14.4] | 4.3[2.2-6.7] | 3.4[2.8-3.9] |
| **FONT** | 12.8[12.1-13.5] | 4.8[2.6-7.3] | 2.9[2.4-3.3] |
| **CEF** | 13.6[12.8-14.5] | 5.4[2.8-8.8] | 2.7[0.9-4.6]] |
| **FAC** | 13.5[12.7-14.2] | 5.1[2.7-7.7] | 3.3[2.63-3.972] |
| **MAS** | 13.7[13.1-14.3] | 4.5[2.2-6.7] | 3.4[2.9-3.8] |
| **BOT** | 13.8[12.6-15.1] | 4.7[2.1-7.5] | 3.2[2.2-4.1] |

**Table S5. Repeatability (rpt), variance components, predicted trait mean and their 95% credible interval for breath rate index, handling aggression and exploration score.** For handling aggression, the within-individual variance is fixed to one due to the family distribution of the model.Note that we do not report the raw variance of among-observer variance in the table, as we are not interested in variance without biological relevance. The among-observer variance represented 6% (95% CI: [1-15%]) of the phenotypic variation in the city and 2% (95% CI: [0-8%]) in the forest for the breath rate index and handling aggression. Additionally, it accounted for 7% (95% CI: [2-14%]) in the city and 14% (95% CI: [5-24%]) in the forest for handling aggression and breath rate index, respectively.

| **metric** | **habitat** | **Breath rate index** | **Handling aggression** | **Exploration Score** |
| --- | --- | --- | --- | --- |
| rpt | urban | \| 0.53[0.42-0.64] \| \| --- \| | \| 0.50[0.39-0.61] \| \| --- \| | \| 0.68[0.56-0.79] \| \| --- \| |
| rpt | forest | \| 0.29[0.12-0.44] \| \| --- \| | \| 0.30[0.17-0.41] \| \| --- \| | \| 0.76[0.62-0.9] \| \| --- \| |
| Vp | urban | \| 5.40[4.44-6.65] \| \| --- \| | \| 3.31[2.38-4.3] \| \| --- \| | \| 3.42[2.76-4.08] \| \| --- \| |
| Vp | forest | \| 6.22[4.35-9.07] \| \| --- \| | \| 2.21[1.6-2.81] \| \| --- \| | \| 7.41[5.46-9.55] \| \| --- \| |
| Vy | urban | \| 0.33[0.03-0.87] \| \| --- \| | \| 0.22[0.05-0.48] \| \| --- \| | \| 0.34[0.02-0.91] \| \| --- \| |
| Vy | forest | \| 0.11[0-0.45] \| \| --- \| | \| 0.21[0.05-0.44] \| \| --- \| | \| 0.59[0-1.6] \| \| --- \| |
| Vf | urban | \| 0.03[0-0.07] \| \| --- \| | \| 0.19[0.09-0.3] \| \| --- \| | \| 0.04[0-0.08] \| \| --- \| |
| Vf | forest | \| 0.07[0-0.17] \| \| --- \| | \| 0.03[0-0.06] \| \| --- \| | \| 0.13[0-0.35] \| \| --- \| |
| Vi | urban | \| 2.83[2.35-3.32] \| \| --- \| | \| 1.67[0.96-2.48] \| \| --- \| | \| 2.32[1.92-2.77] \| \| --- \| |
| Vi | forest | \| 1.73[0.93-2.59] \| \| --- \| | \| 0.66[0.3-1.07] \| \| --- \| | \| 5.65[3.89-7.52] \| \| --- \| |
| Vr | urban | \| 1.68[1.41-1.95] \| \| --- \| | Fixed to 1 | \| 0.72[0.54-0.94] \| \| --- \| |
| Vr | forest | \| 2.64[1.91-3.44] \| \| --- \| | Fixed to 1 | \| 1.04[0.38-1.89] \| \| --- \| |
| Predicted mean | urban | 13.44[12.72, 14.07] | 2.01[1.46, 2.58] | 3.02[2.64, 3.51] |
| Predicted mean | forest | 14.20[13.24, 15.19] | 1.89[1.37, 2.47] | 1.28[0.60, 1.88] |

**Table S6. Log ratio of repeatability (A, lnRPT), coefficient of variation (A, lnCVR), and average behavior (B, lnRR) for the three behavioral traits, along with their corresponding 95% credible intervals**. LnRPT, lnCVR, and lnRR with credible intervals that do not overlap zero are represented in bold. Note it was not possible to estimate residual variation of handling aggression.

| .**A -Trait** | **Log ratio for behavioral variation** | **effect size** |
| --- | --- | --- |
| Breath rate index | lnRPT | **0.42[0;0.95]** |
| Breath rate index | lnCVR Among-individual | **0.3[0.03;0.6]** |
| Breath rate index | lnCVR year | 0.94[-0.84;3.74] |
| Breath rate index | lnCVR fixed effects | -0.34[-1.51;0.95] |
| Breath rate index | lnCVR residual | -0.17[-0.35;0.02] |
| Breath rate index | lnCVR phenotypic variance | 0.09[-0.05;0.24] |
| Handling aggression | lnRPT | **0.45[0.18;0.81]** |
| Handling aggression | lnCVR Among-individual | **0.41[0.06;0.8]** |
| Handling aggression | lnCVR year | -0.04[-0.83;0.78] |
| Handling aggression | lnCVR fixed effects | **0.9[0.22;1.7]** |
| Handling aggression | lnCVR phenotypic variance | **0.24[0.12;0.35]** |
| Exploration score | lnRPT | -0.12[-0.41;0.18] |
| Exploration score | lnCVR Among-individual | **-1.3[-2.08;-0.8]** |
| Exploration score | lnCVR year | -1.13[-2.4;0.26] |
| Exploration score | lnCVR fixed effects | **-1.48[-2.83;-0.15]** |
| Exploration score | lnCVR residual | -0.14[-0.57;0.23] |
| Exploration score | CV phenotypic variance | **-1.09[-1.26;-0.91]** |
| **B -Trait** | **Log ratio for average behavior** | **effect size** |
| Breath rate index | lnRR male | -0.05[-0.14-0.04] |
| Breath rate index | lnRR female | -0.05[-0.15-0.03] |
| Breath rate index | lnRR adults | -0.05[-0.14-0.04] |
| Breath rate index | lnRR juveniles | -0.05[-0.14-0.03] |
| Handling aggression | lnRR male | 0.19[-0.11;0.47] |
| Handling aggression | lnRR female | -0.16[-0.58;0.2] |
| Handling aggression | lnRR adults | 0.07[-0.25;0.38] |
| Handling aggression | lnRR juveniles | 0[-0.34;0.34] |
| Exploration score | lnRR male | **1.17[0.55;2.71]** |
| Exploration score | lnRR female | **0.78[0.27;1.55]** |
| Exploration score | lnRR adults | **1.08[0.53;2.23]** |
| Exploration score | lnRR juveniles | **0.87[0,31:1,67]** |


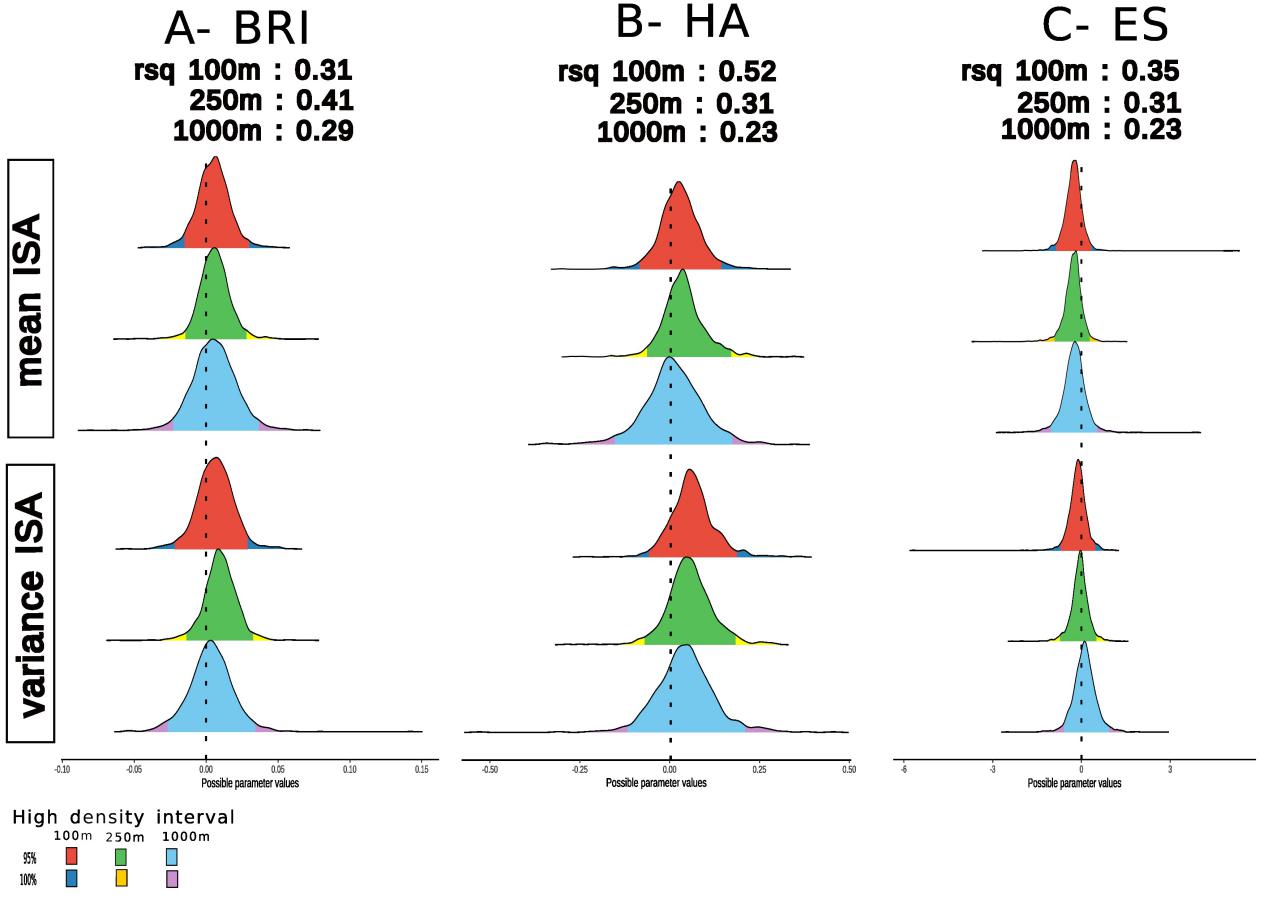


**Figure S2. Posterior distribution of the magnitude of relationship between mean ISA and variance in ISA with the mean-standardized total phenotypic variance (CVP), for breath rate index (BRI) handling aggression (HA) and exploration score (ES).** High density interval 95% is reported in red, green, and light blue for ISA measured at 100, 250 and 1000m respectively. High density interval 100% is reported in dark blue, yellow and purple for 100m, 250m and 1000m respectively. Rsq refers to the median of bayesian-R2 over the iterations of the models and the highest rsq is reported in bold.

**
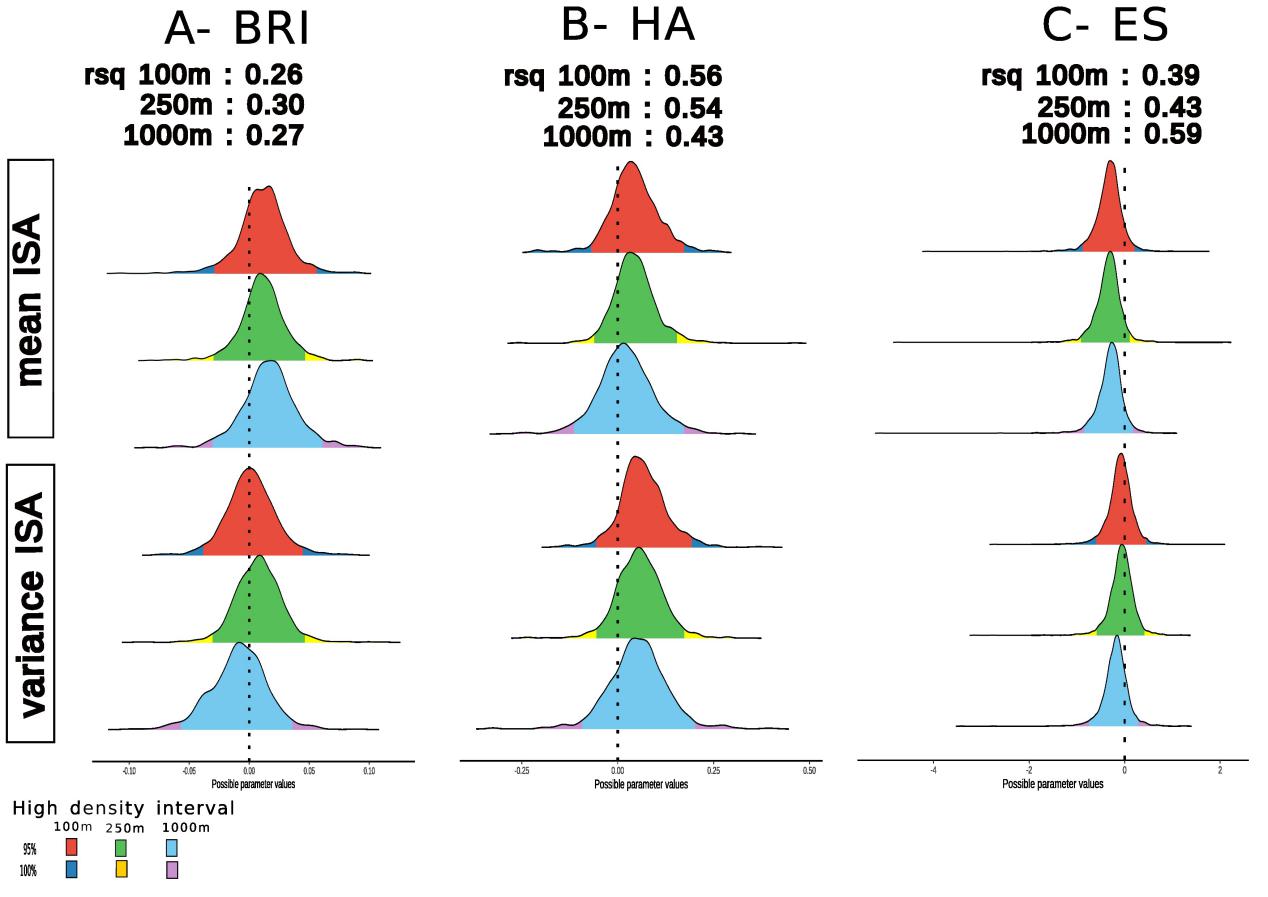
Figure S3. Posterior distribution of the magnitude of relationship between mean ISA and variance in ISA with the mean-standardized among-individual variance (CVI), for breath rate index (BRI) handling aggression (HA) and exploration score (ES).** High density interval 95% is reported in red, green, and light blue for ISA measured at 100, 250 and 1000m respectively. High density interval 100% is reported in dark blue, yellow and purple for 100m, 250m and 1000m respectively. Rsq refers to the median of bayesian-R2 over the iterations of the models and the highest rsq is reported in bold.


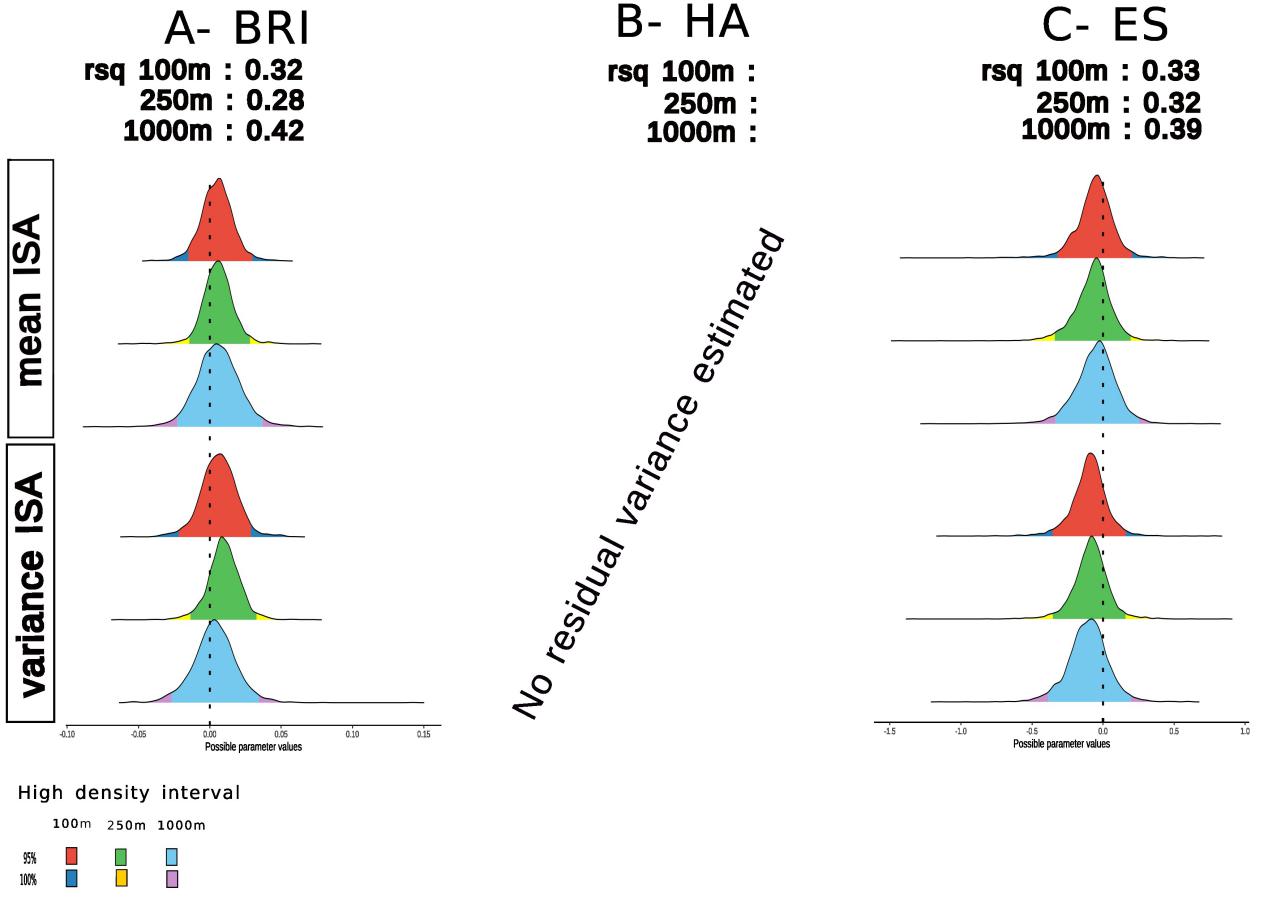


**Figure S4. Posterior distribution of the magnitude of relationship between mean ISA and variance in ISA with the mean-standardized within-individual variance (CVR), for breath rate index (BRI) and exploration score (ES).** High density interval 95% is reported in red, green, and light blue for ISA measured at 100, 250 and 1000m respectively. High density interval 100% is reported in dark blue, yellow and purple for 100m, 250m and 1000m respectively. Rsq refers to the median of bayesian-R2 over the iterations of the models and the highest rsq is reported in bold. Note that because we used a threshold model it was not possible to estimated residual variance.


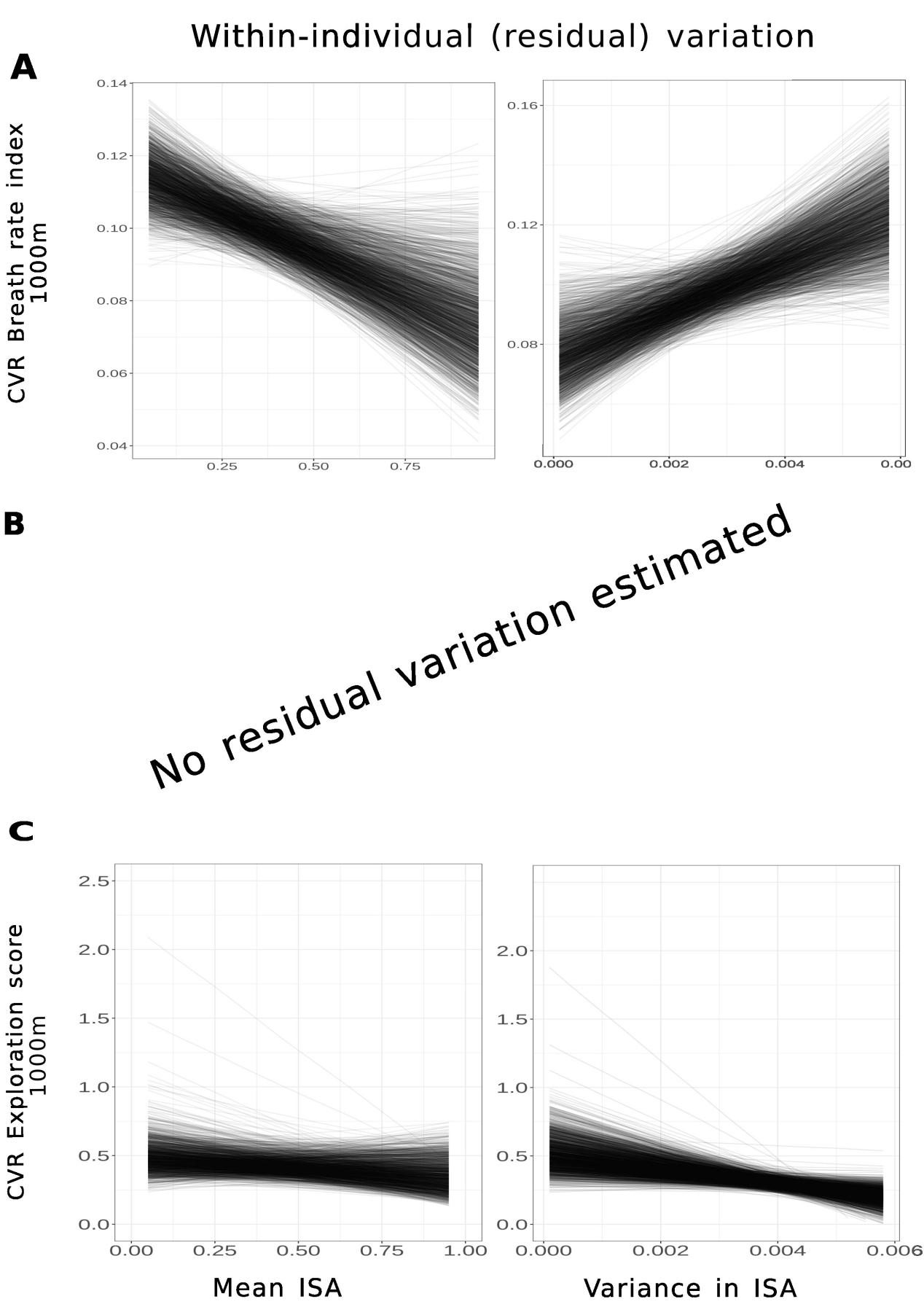


**Figure S5. Relationship between predicted mean-standardized residual variance (CVR) and mean ISA (on the left), variance ISA (on the right) for Breath Rate Index (BRI, A), Handling aggression (HA, B) and exploration score (ES, C) and their corresponding «scale effect».** CVR used here are estimated from the posteriors distributions of model b. Each line represents the predictions of one Bayesian model (one model per iteration of model b). Note that CVR is expressed on the latent-scale for exploration score and no CVR was estimated for handling aggression because of the statistical model.


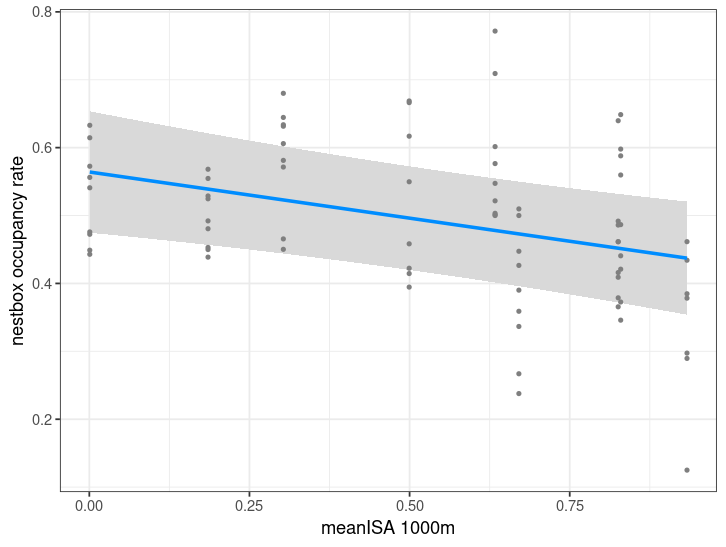


**Figure S6. Relationship between nestbox occupancy rate and the average impervious surface assessed in a buffer of 1000m radius.**
